# ‘Skip’ osteoporosis vertebral compression fractures caused by electrical injury: a case report and review of the literature

**DOI:** 10.1186/s13256-024-04358-w

**Published:** 2024-02-14

**Authors:** Ruili Jia, Yanhao Sun, Chong Liu, Rui-chao Liu, Yubin Long

**Affiliations:** 1 The Department of Nephropathy, Baoding First Central Hospital, Baoding, Hebei People’s Republic of China; 2 The Third Department of Orthopedics, Baoding First Central Hospital, Baoding, Hebei 071000 People’s Republic of China; 3 The Department of Radiology, Baoding First Central Hospital, Baoding, Hebei People’s Republic of China

**Keywords:** Osteoporosis, Vertebral compression fractures, Electrical injury, Young

## Abstract

**Introduction:**

Electrical injuries rarely result in fractures, such as long bone fractures and spinal fractures. A few articles have reported osteoporosis vertebral compression fractures (OVCFs) caused by electrical injuries. Here, we present a rare case of 37-year-old male suffering from the 9th thoracic (T9) and 5th lumbar (L5) OVCFs after receiving a electric shock.

**Case presentation:**

A 37-year-old Han male experienced an electric shock (480 V direct current) at the working time and felt immediately serious back pain. He did not fall and lose consciousness. X-ray and magnetic resonance imaging showed acute OVCFs, as well as dual-energy X-ray absorptiometry indicated osteoporosis. Normal laboratory tests can avoid secondary osteoporosis resulting from metabolic diseases and tumors. Finally, he was diagnosed with acute discontinuous OVCFs (T9 and L5). The patient denied having a history of back pain, whereas, he had a history of smoking, alcohol abuse, and congenital heart disease (tetralogy of Fallot) were associated with osteoporosis. Considering no local kyphosis and < 50% anterior body compression, we selected conservative treatment for this patient. At a 1-year and 3-year follow-up, the lateral thoracic and lumbar radiography demonstrated no instability of the spine, and the back pain has been relieved.

**Conclusions:**

This rare case reminds us the importance of consulting a detailed medical history when we encounter young patients receiving electrical injuries. Discontinuously OVCFs must not be overlooked, even though we encounter a young man.

## Introduction

Fractures caused by electric shock are not very common. According to previous articles [1, 2], the most common fractures after electrical injuries were posterior fracture dislocation of the humeral head, humeral neck fractures, or scapular fractures, while vertebral fractures were extremely rare [3, 4], especially vertebral compression fractures (VCFs). Osteoporosis vertebral compression fractures (OVCFs), the most common osteoporotic fracture in the aging population, commonly lead to severe pain among symptomatic patients [5], which is often induced by minimal trauma such as low-grade fall or slip down injury [6]. Regarding vertebral fractures, Wimar [3] showed a rare case of lumbar burst fracture due to low voltage shock, and Sinha [4] presented a case of thoracic compression fracture caused by electrically induced injury. Furthermore, in terms of OVCFs, to our best knowledge, only a case has been reported by Jeon [7] in which a 76-year old female patient suffered from acute OVCF at the thoracic spine after using an electrical automated massage chair. In clinical practice, the treatment can be divided into surgical treatment and conservative treatment, including nonpharmacological and pharmacological methods, such as supplementary calcium, vitamin D, anti-resorptive agents, hormone therapy, and anabolic agents [8].

To our knowledge, there has been no case report focusing on discontinuous OVCFs caused by electrical injuries. We present a rare case of a 37-year-old man with the 9th thoracic and 5th lumbar OVCFs as a result of an electrical injury.

### Ethics approval and consent to participate

The study was approved by the institutional review board of our hospital before data collection and analysis. There is no need to write informed consent forms from patients because this is a retrospective study.

## Case presentation

A 37-year-old Han man with a dominant right hand, a factory worker with a history of smoking, alcohol abuse, and receiving heart surgery due to congenital heart disease (tetralogy of Fallot), was hospitalized after an electric shock (480 V direct current). He suffered from an electrical shock while preparing to change the wires of a machine. According to the statement of the patient and eyewitness (coworkers), this patient drew back his left hand instantaneously and then tossed it into the arms of his colleagues, feeling immediately serious back pain. The patient recalled that his back was forcefully contracted and stretched excessively at the time of the injury. He did not fall and lose consciousness during the accident.

**Fig. 1:**
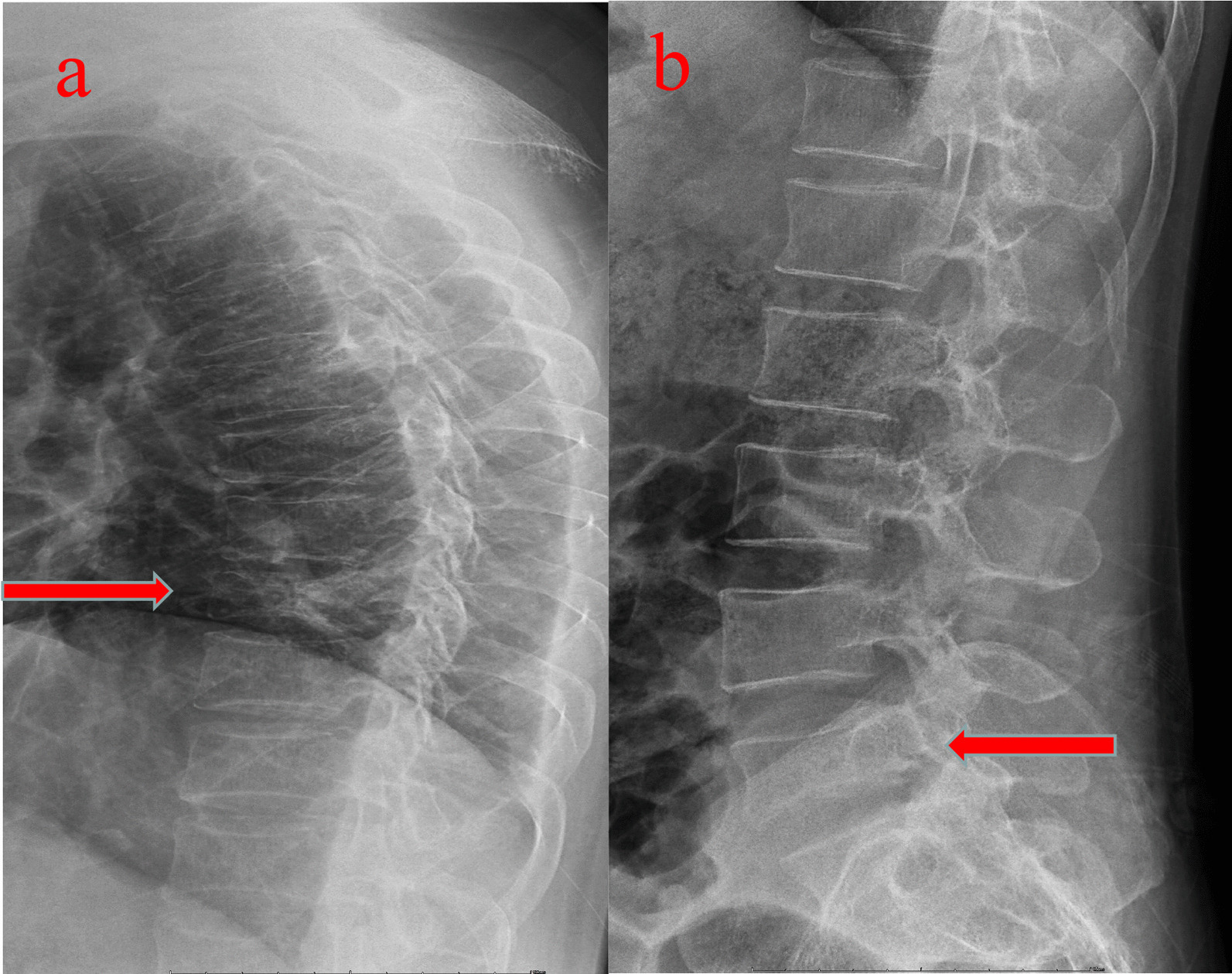
Radiology date of the patient at the time of injury. **a** The lateral X-ray showed 9th thoracic (T9) vertebral compression fractures (VCFs). **b** The lateral X-ray showed 5th lumbar (L5) VCFs. Arrows showed the T9 and L5 fracture

**Fig. 2:**
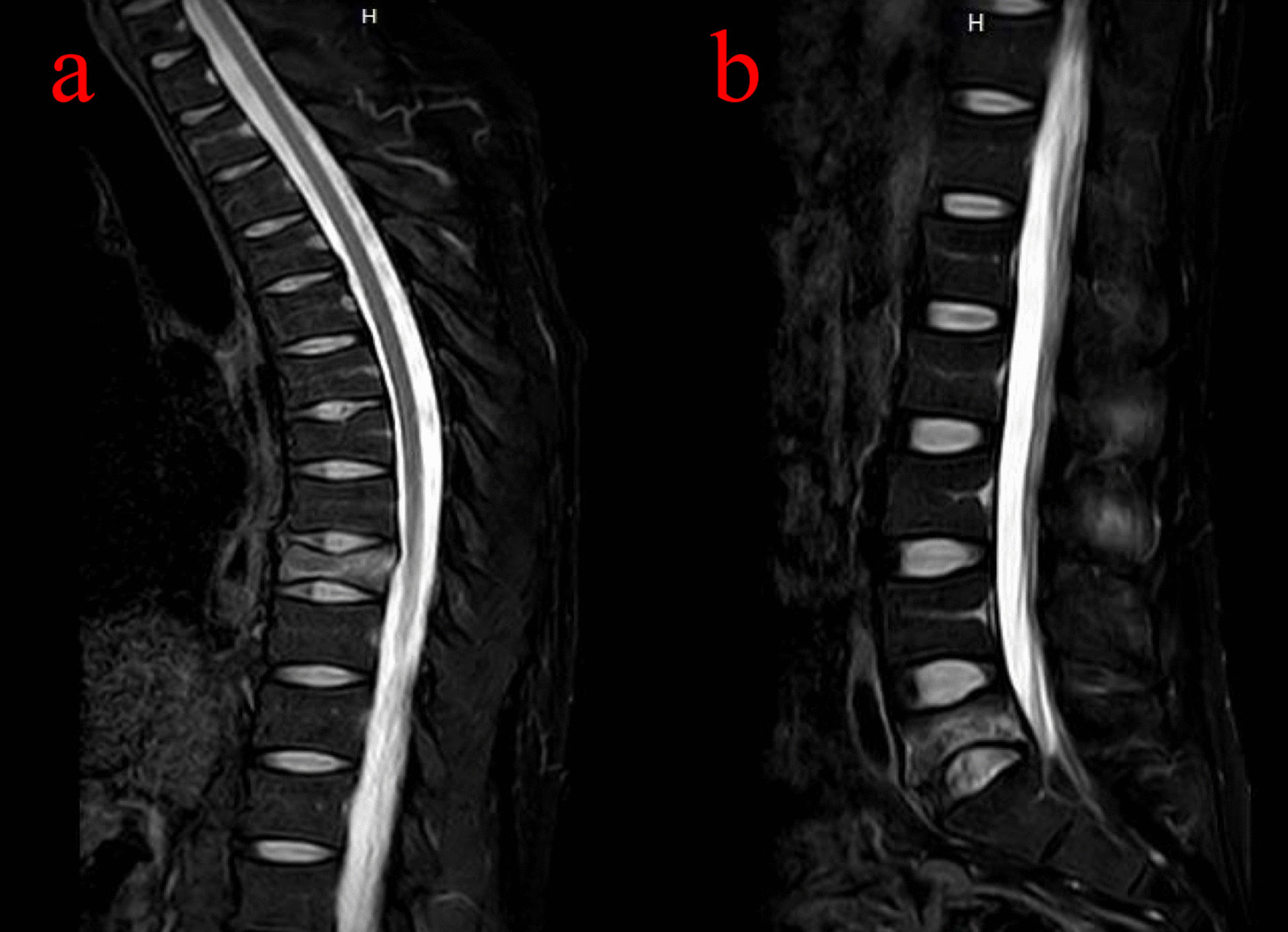
Radiology date of the patient at the time of injury. **a** Magnetic resonance imaging (MRI) showed T9 VCFs. **b** MRI showed L5 VCFs

**Fig. 3:**
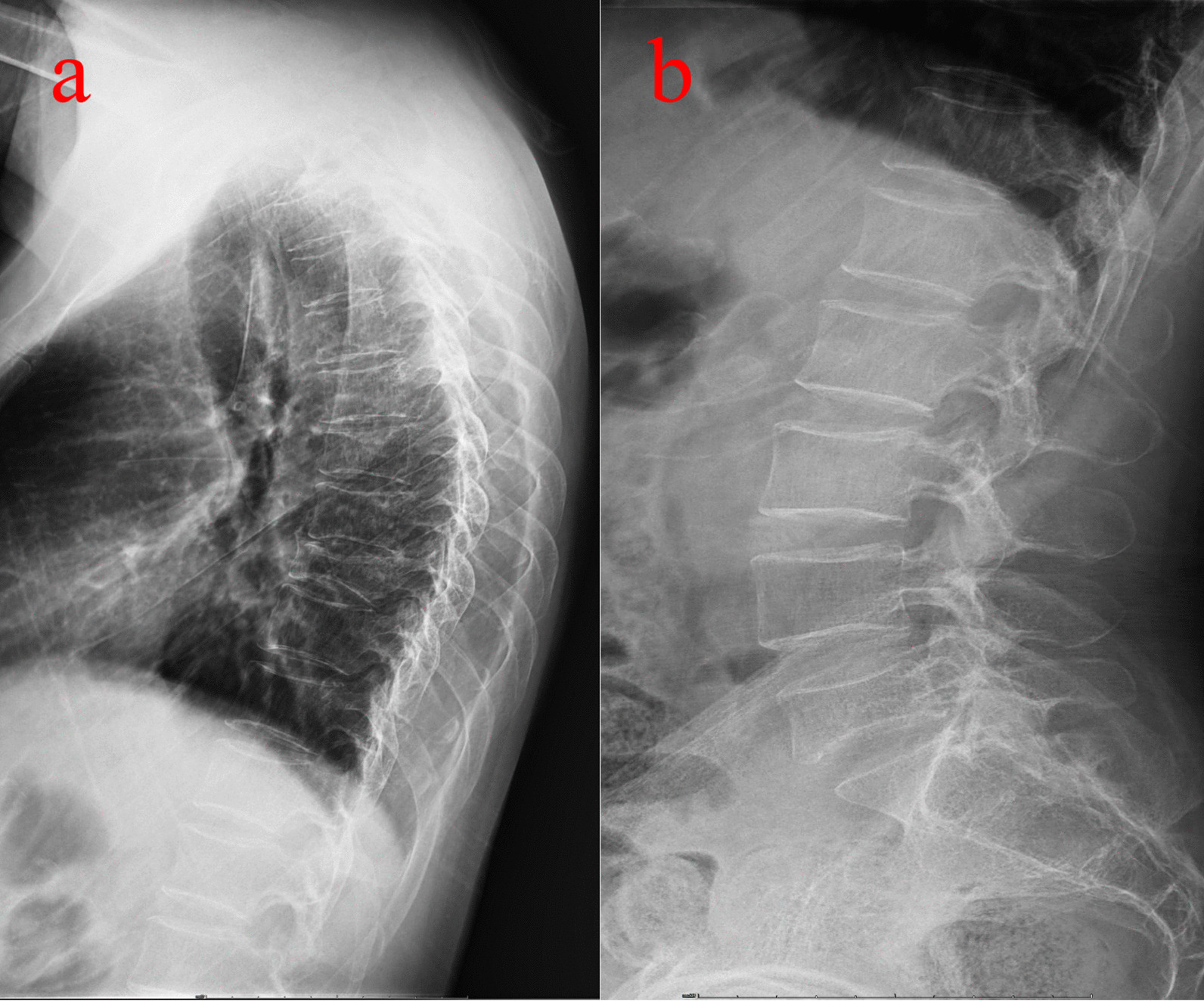
Radiology date of the patient at 1-year follow-up. **a** The lateral X-ray showed showed T9. **b** The lateral X-ray showed L5

**Fig. 4:**
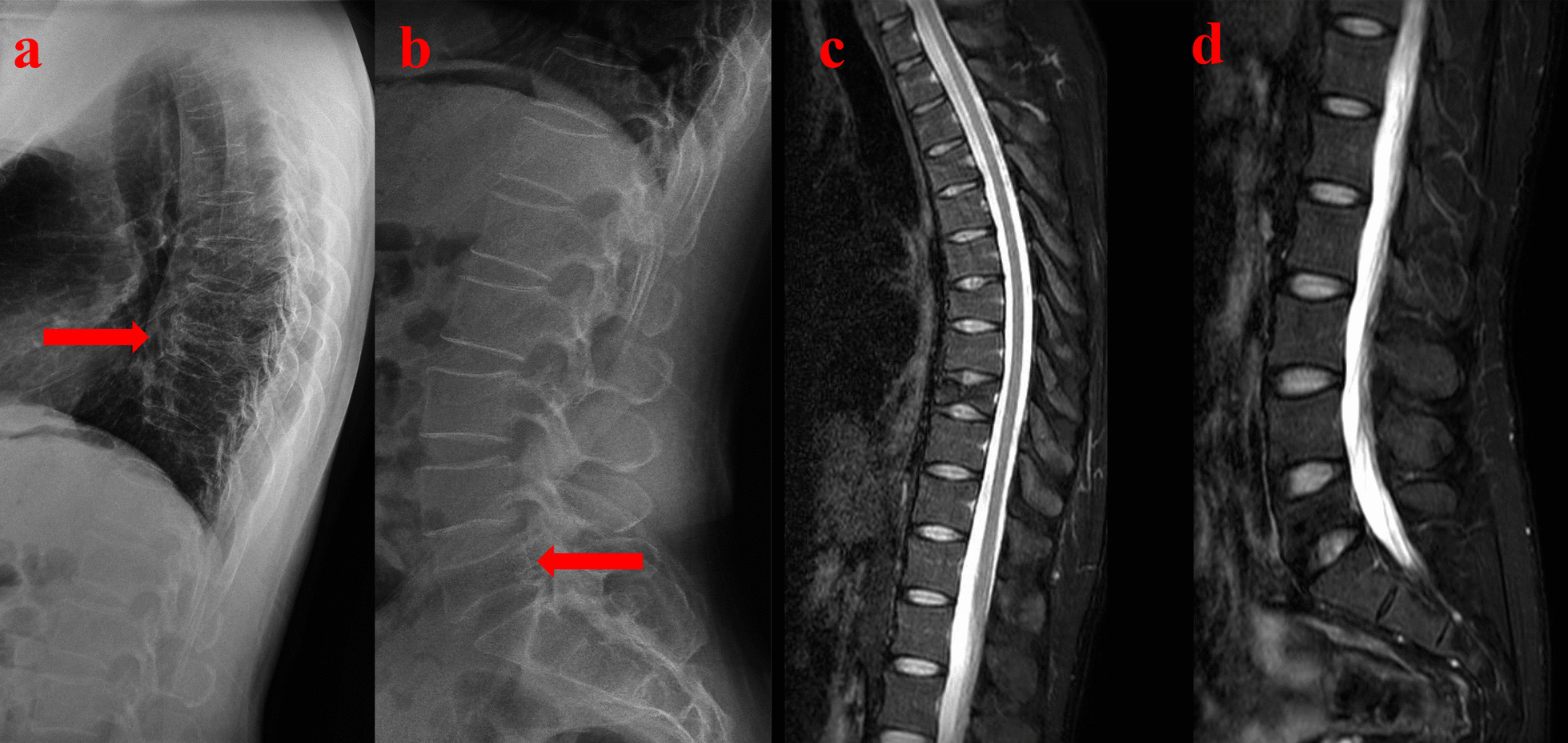
Radiology date of the patient at 3-year follow-up. **a** The lateral X-ray showed showed T9. **b** The lateral X-ray showed L5. **c** Magnetic resonance imaging (MRI) showed T9 VCFs. **d** MRI showed L5 VCFs. Arrows showed the T9 and L5 fracture

Physical examination revealed restricted back mobility, discomfort, and percussion pain in the 9th thoracic (T9) and 5th lumber (L5) areas. Functions (sensory and motor) of all peripheral nerves were tested and were normal. He denied chest pain, palpitations, shortness of breath and the history of back pain. Lateral X-rays of the thoracic and lumber regions revealed T9 and L5 vertebral compression fractures (VCFs) (Fig. 1a, b), and magnetic resonance imaging (MRI, Fig. 2a, b) revealed acute VCFs (T9 and L5) which were commonly caused by osteoporosis [6]. Surprisingly, it was worth noting that the young patient suffered from osteoporosis, which was proved by dual-energy X-ray absorptiometry (DXA) measurement (T score was less than − 2.5 in the spine, femoral neck, and 1/3 radius). Other normal laboratory tests can avoid secondary osteoporosis caused by metabolic diseases and tumors. Finally, this patient was diagnosed with acute discontinuous OVCFs (T9 and L5). We chose anti-osteoporosis therapy, such as supplementary calcium, vitamin D, and anti-resorptive agents because there was no local kyphosis and < 50% anterior body compression. At 1-year follow-up, back pain was relieved and an X-ray showed no local kyphosis and less than 50% anterior body compression, but the height of the vertebral column was still lost, as shown in Fig. 3a, b. The height of the vertebral column generally recovers at 3-year follow up (Fig. 4a–d) and bone mineral density was − 2.5 in the spine, femoral neck, and 1/3 radius by DXA.

## Discussion

Electrical injuries, which cause tissue destruction and organ dysfunction, are a typical occurrence in emergency departments [8]. However, fractures, especially vertebral fractures, are uncommon. After reviewing related articles, Stone N [9] came to the conclusion that proximal humerus and scapular fractures were the most common fractures after an electrical shock. A 20-year-old man suffered a rare case of lung injury and two fractures in the arches of the C1 vertebrae after an accidental high-voltage electrocution [10]. Wimar [3] was the first to report a 62-year-old man who had a lumbar burst fracture as a result of a low-voltage electrical shock. Sinha [4] described a case of a thoracic compression fracture caused by electricity. A case of two thoracic compression fractures due to a shock from a conducted energy weapon was also reported by Winslow [11]. The thoracolumbar junction is the most commonly involved area for OVCFs [12], while low lumbar fractures account for 1.2% to 2% of all spinal injuries [13–15]. To our knowledge, no literature has described ‘Skip’ OVCFs. As far as we know, this was the first case of a 37-year-old man experiencing discontinuous OVCFs (T9 and L5) after receiving an electrical shock without falling.

There were three possible explanations for our findings. First, the osteoporosis in this patient played a crucial role in developing VCFs. Amazingly, it was atypical for a 37-year-old man with osteoporosis. What was the best way to explain this phenomenon? After excluding some causes of secondary osteoporosis, such as metabolic diseases and tumors, we combed through his medical records and discovered possible risk factors such as his work schedule (night shift work) and a history of smoking, drinking, and the tetralogy of Fallot. Night shift work may have an indirect influence on bone physiology, which might be a risk factor for osteoporosis [16]. Smoking and alcohol abuse, both of which this patient had, were substantial risk factors for osteoporosis [17–19]. In addition, individuals with complicated congenital heart disease had lower overall bone mineral density than healthy controls [20]. The abovementioned might be important factors related to the patient with osteoporosis. Second, bone has the largest resistance of any biological tissue, implying that when exposed to an electrical current, it creates the most heat or energy, which may lead to fracture. Third, forceful muscle contraction due to electric shock may contribute to T9 and L5 VCFs. According to previous studies [3, 4, 10, 11], the mechanism of fracture after electrical injury might be associated with forceful muscle contraction. As an example, the infraspinatus and teres minor greatly contracted along with the deltoid, latissimus dorsi, and teres major, forcing the humeral head superiorly and posteriorly against the acromion and medially against the glenoid fossa, which caused the humeral head to lodge behind the glenoid rim [2]. Similarly, strong contraction of back muscles due to electrical injury may lead to the fracture of the T9 and L5 VCFs.

To our knowledge, discontinuous OVCFs across seven segments have not been reported in patients with falling, let alone this patient receiving an electric shock. Due to its unique anatomical and biomechanical features, such as its position below the pelvic brim and the peak of the lumbar lordosis, as well as the stabilizing impact of the iliolumbar ligaments that protect this area from severe damage, a low lumbar vertebra fracture is an uncommon entity. Butler [15] described 14 cases of L5 burst fractures caused by axial compressive pressures during flexion. To our knowledge, this was the first study to describe the L5 OVCFs. We inferred that the great contraction of back muscles and energy generated by electrical current caused the L5 OVCFs. However, we did not understand the underlying mechanism of discontinuous OVCFs, which needs to be studied in the future.

Osteoporotic fracture prevention necessitates the identification of patients at risk and the use of both pharmacological and nonpharmacological treatments to reduce such risk factors [21]. So far, several clinical risk factors have been discovered [22]. A variety of imaging modalities are used to assess bone condition in terms of density and quality, as well as biochemical markers. Bone metabolism studies have also been conducted. Various medications interfere with bone metabolism, especially calcium Vitamin D, anti-resorptive therapy, hormone therapy, and other treatments with anabolic steroids [8]. We chose anti-resorptive therapy for this patient because there was no local kyphosis and 50% anterior body compression, and no back discomfort was reported at 1-year follow-up, indicating that conservative therapy was also an effective method at 1-year follow-up. Loss of vertebral height still existed in the X-ray of the 1-year follow-up, but there was general recovering at the 3-year follow-up. Bone mineral density is recovery from − 2.5 at the time of injury to − 1.0 at 3-year follow-up. What we are concerned about is facet degeneration and adjacent vertebral disease, or even local kyphosis in a longer follow-up. Thus, a longer follow-up must be performed to observe changes in the curvature of the spine and recover vertebral height.

## Conclusions

This rare case reminds us of the importance of consulting a detailed medical history, including their history, living habits, working habits, and environment, when we encounter young patients receiving electrical injuries. Fractures, especially discontinuous OVCFs as present in this case, must not be overlooked, even though we encounter a young man. Furthermore, our outcomes at 3-year follow-up demonstrate the efficacy of anti-osteoporosis therapy.

## Data Availability

Yes.
